# Skin Closure Tape and Surgical Staples in Primary Total Knee Arthroplasty: A Systematic Review and Meta-Analysis

**DOI:** 10.1155/2020/4827617

**Published:** 2020-01-10

**Authors:** Xiangli Luo, Wenhui Zhang, Peijing Yan, Zongru He, Yuping Yang, Kehu Yang, Yaowen Qian

**Affiliations:** ^1^Department of Orthopedics, Gansu Provincial Hospital, Donggang West Road, Lanzhou 730000, Gansu, China; ^2^School of Clinical Medical Sciences, Ningxia Medical University, Shengli Street, Yinchuan 750000, China; ^3^Institute of Clinical Research and Evidence Based Medicine, Gansu Provincial Hospital, Lanzhou 730000, China; ^4^Evidence-Based Medicine Center, School of Basic Medical Sciences, Lanzhou University, Lanzhou 730000, China; ^5^Evidence Based Social Science Research Center, Lanzhou University, Lanzhou 730000, China; ^6^Key Laboratory of Evidence Based Medicine and Knowledge Translation of Gansu Province, Lanzhou 730000, China

## Abstract

**Background:**

Staples closure technology has been widely used in total knee arthroplasty (TKA) and achieved good results. In recent years, a new type of material called skin closure tape (SCT) has been applied to TKA which also showed good treatment results. However, since it is still not clear yet which one is better, this paper collects literatures for statistical analysis so as to provide evidence for the use of SCT in TKA.

**Methods:**

The comparative study on effects between SCT and staples is reviewed after the primary release of TKA in PubMed, the Cochrane library, and the EMBASE database up to March 2019. The two researchers independently screened the literature and evaluated the quality of the literature using bias risk tools.

**Results:**

A total of four studies (3330 knees) have been included in our meta-analysis. For the main point, the results show that the SCT can reduce readmission rates compared to staples (RR 0.68, 95% CI 0.49–0.95, *P*=0.03), with no significant difference in complications (RR 0.85, 95% CI 0.27–2.64, *P*=0.77). Secondly, the results suggest that although there is no significant difference in removal time between the two groups, the SCT can reduce pains, save time and costs, and have a better cosmetic effect.

**Conclusions:**

Our study indicates SCT as a closure method with fewer complications and faster speed compared with staples. Nevertheless, the cost and pain need to be further confirmed because of the small sample size included in this study.

## 1. Introduction

Along with the gradual aggravation of population aging, the incidence of knee disease is also gradually increasing, leading to the increasing number of people receiving total knee arthroplasty (TKA) [1, 2]. TKA, as one of the important treatments for the end-stage knee disease, can effectively correct and relieve pain and improve the life quality of the patients [3]. Being an important part of TKA, good skin closure techniques can significantly prevent infections and increase patient satisfactions [4, 5]. Currently, the skin closure method commonly used by doctors includes two methods of traditional suture and surgical staples. A systematic and meta-analysis, published in 2017, compared the safety and effectiveness of staples in primary total knee arthroplasty [6] It indicated lower wound complications, decreased wound closure time, and an overall reduction in resource utilization of staples, which expressed the same idea with Krishnan et al. [7].

In recent years, a new noninvasive technique of skin closure called skin closure tape (SCT) has emerged and has been applied to clinical practices [8]. As some studies have pointed out the advantages of the new technology over Staples [9], such as easy apply, less pain, better cosmetic outcomes, and so on, we, however, still query all the databases and find no systematic review and meta-analysis concerning SCT and staples. Therefore, our meta-analysis and systematic review will be the first research to comprehensively compare the applications of SCT and staples in TKA.

In addition, given the high quality, meta-analysis has been increasingly regarded as one of the key tools for collecting evidence [10–12]. We performed a meta-analysis to assess SCT and staples in patients undergoing primary TKA and to obtain evidences for the applications of SCT in the primary TKA.

## 2. Methods

### 2.1. Search Strategy

The current study is conducted according to the Preferred Reporting Items for Systematic Reviews and Meta-Analyses guidelines [10, 13]. A MeaSurement Tool to Assess Systematic Reviews (AMSTAR) is employed to evaluate the methodological quality of systematic review [14, 15]. The Newcastle-Ottawa Quality Assessment Scale is adopted for assessing the quality of the included study.

Two researchers independently review literatures published on multiple databases including EMBASE, PubMed, and Cochrane Library up to March 2019. The key word which can be retrieved together with free word for literature inquiry is imputed as “knee arthroplasty” or “knee replacement” or “joint arthroplasty” or “joint replacement” or “TKA” or “TKR” and “skin closure tape” or “suture-free tape” or “adhesive tape” or “wound closure tape” or “steri-strip” or “tape” and “staple” or “U-type nail” or “metal pin” and so on, when the references of the identified studies are manually searched with no limitation on year or language.

### 2.2. Selection Criteria

The inclusion criteria are listed as follows: (a) patients in the study received primary TKA treatment without being interfered with the clinical results of the remaining related diseases; (b) the study reports the comparison between SCT and staples; and (c) at least one of the following should be present: ① wound complications, ② readmission rates (total readmission rates and readmission rates of wound-related), ③ time to removal, ④ costs (total costs and material costs), ⑤ Vancouver scar score (VSS), and ⑥ Visual analogue scale (VAS).

The exclusion criteria are as follows: (a) duplicate articles; (b) case reports, reviews, meta-analysis, editorials, letters, non-English, nonhuman, and cadaver experimental studies; (c) data that could not be extracted; and (d) reports that are not relevant to this study.

### 2.3. Date Extraction

Two researchers independently extract data from the included studies: first author's name, date of publication, country, average age of the patient, sample size, patients' genders, body mass index (BMI), follow-up time, and key indicators of ①wound complications and ② readmission rates and secondary indicators including ③ time to removal, ④ costs, ⑤ Vancouver scar score (VSS), and ⑥ Visual analogue scale (VAS). In the case of data loss, we try to contact the corresponding authors for details, and if the two researchers disagree, we seek for the help from a third researcher.

### 2.4. Statistical Analysis

The RevMan 5.3 software provided by the Cochrane Collaboration Network is applied to conduct the statistical analysis, and the heterogeneity between the studies uses Q-test and *I*^2^ test. ① If *P* > 0.1 or *I*^2^ ≤ 50%, we think there is no obvious heterogeneity between the included studies and then we use the fixed-effect model to merge the data. ② If *P* < 0.1 or *I*^2^ > 50%, we consider that a heterogeneity exists among many results and the random effect model will be used to combine data and analyze heterogeneous sources. For continuous variables, we use mean difference (MD) with 95% confidence interval (95% CI) and risk ratios (RR) with 95% confidence interval (95% CI) for classification variables. The test level was set to 0.05, and the heterogeneity of clinical manifestations was analyzed by grouping analysis or sensitivity analysis, or only descriptive analysis.

## 3. Results

### 3.1. Study Selection

**Figure 1:**
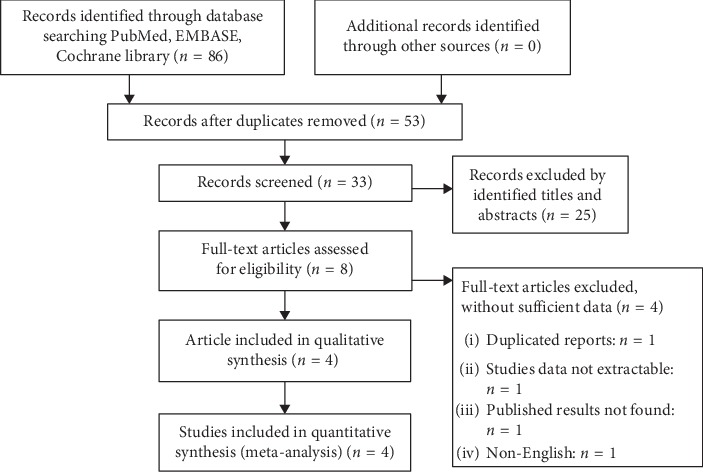
Flowchart of the literature search in the meta-analysis.

Through systematic retrieval on the EMBASE, PubMed, and the Cochrane library, a total of 86 studies are obtained with a total of 53 articles being excluded because of duplications. After primarily reading the article titles and abstracts, 25 articles are then excluded due to lack of obvious correlations and further exclude 4 articles after reading the full-text details (① duplicated studies: *n* = 1, ② studies with unextractable data: *n* = 1, ③ published results that cannot be found: *n* = 1, and ④ non-English: *n* = 1). The last 4 articles meet all the criteria are included in the analysis, and our literature search process is shown in Figure 1.

### 3.2. Characteristics of the Included Studies

The study is conducted during the period from 2017 to 2018, for a total of 4 studies [8, 9, 16, 17] with 3330 knees (1275 knees for males and 2055 knees for women). All the basic information needed of inclusion literature is shown in Table 1.

### 3.3. Outcome Analysis

#### 3.3.1. Meta-Analysis

**Figure 2:**
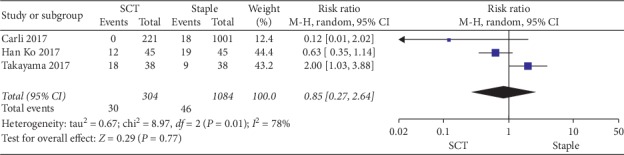
Forest plot on the assessment of wound complications.

*(1) Wound Complications*. Three studies included wound complications [8, 9, 17], with no significant difference detected regarding complications between the two groups (RR 0.85, 95% CI 0.27–2.64, *P*=0.77); the forest diagram of wound complications is shown in Figure 2.

**Figure 3:**
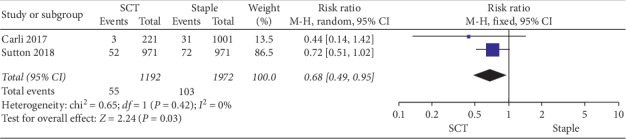
Forest plot on the assessment of readmissions.

*(2) Readmission Rates*. Two studies presented the total readmission rates [8, 16], with one study reporting wound-related readmission rates [8], and there was no significant heterogeneity among the subgroups (*P*=0.42, *I*^2^ = 0%). The rest two studies included showed significantly lower total readmission rates with SCT than staples (RR 0.68, 95% CI 0.49–0.95, *P*=0.03); the forest diagram of readmission rates is shown in Figure 3.

#### 3.3.2. Systematic Review

*(1) Time to Removal*. According to the content about the time to removal in one study [9], the authors, Han Ko et al. [9] reported the shorter time to removal of the SCT compared to that of the staples (13.89 ± 0.70 day vs 14.00 ± 0.88 day). However, this difference has no statistical significance (*P*=0.512).

*(2) Costs*. As it was reported in one study, Sutton et al. [16] suggested lower total costs of the SCT than that of the staples ($15593 ± 5.985 vs $16794 ± 9.372). Two studies reporting the material costs [8, 17]to be lower for the SCT compared to that of the staples ($3 vs $26) were explored by Takayama et al. [17], but Carli et al. [8] thought that the material costs were higher for the SCT than that for the staples ($63 vs $45).

*(3) Vancouver Scar Score*. There is one study that reported the VSS [9]. Han Ko et al. [9] reported that the VSS for cosmetic outcome on postoperative day 90 was significantly better in the SCT compared to that in the staples (4.6 ± 0.7 vs 6.9 ± 1.3, *P*=0.043).

*(4) Visual Analogue Scale*. One study reported the VAS [9]. Han Ko et al. [9] reported that the VAS of SCT was significantly lower compared to that of the staples on postoperative 1, 3, and 14 days (3.5 ± 1.0 vs 5.4 ± 0.9, *P* < 0.01; 2.4 ± 0.6 vs 3.6 ± 0.8, *P* < 0.01; and 1.9 ± 1.7 vs 6.4 ± 1.8, *P* < 0.01).

*(5) Sensitivity Analysis*. Our research includes case-control studies and cohort studies. To test the stability of the results of our study, we carried out the sensitivity analysis of main outcome indicators. The process is as follows: for the main outcome indicators for wound complications, we randomly eliminate one of the indicators included in the study, and the obtained results are consistent with the original results. Then, eliminating another indicator, we obtained the same results and the results are consistent with the original results; therefore, sensitivity test results show that the results of our research are less sensitive and have good stability.

## 4. Discussion

TKA is one of the treatments for end-stage knee joint disease, and skin closure is an important part of TKA. If the wound does not close properly or the closure technique is not appropriately conducted after a surgery, some complications may occur, for example, superficial infection [18]. Therefore, the selection of an appropriate wound closure technique is crucial. There are some meta-analysis assessments about the different outcomes of wound closure using different techniques after TKA. Meena et al. [19] reported that the application of barbed suture after the primary TKA could achieve better results than traditional suture, but this study mainly focused on the joint capsule and subcutaneous. Kim et al. [6] conducted a meta-analysis and systematic review to compare staples to traditional sutures concerning skin closure after primary TKA, and the study demonstrated that staples had lower wound complications, decreased wound closure times, and so on. Krishnan et al. [7] also reported that staples could effectively reduce closure time compared to traditional suture. However, to our knowledge, no meta-analysis and systematic review has compared staples and SCT after primary TKA.

In terms of wound complications, Takayama et. al. [17] reported that the wound complications were higher in the SCT compared to staples (47.37% vs 23.68%), but Han Ko et al. [9] reported that the wound complications were lower in the SCT compared to that of the staples (26.67% vs 42.22%). Furthermore, Carli et al. [8] reported significantly lower wound complications with SCT than staples (*P*=0.045). Our meta-analysis demonstrates that there is no significant difference detected in complications between the two groups. According to our experience, the possible causes of different wound complications in different studies are as follows: ① we believe that different clinicians may have different causes of complications due to different closure techniques; ② in different countries or hospitals, different caring levels may lead to different results of complications.

Additionally, we discuss about the readmission rates from two aspects including all-cause/total hospital readmission rates and wound-related hospital readmission rates in 90 days after surgery. ① For all-cause hospital readmission rates, Carli et al. [8] reported that SCT had lower readmission rates than staples (1.36% vs 3.09%), but this difference was not statistically significant (*P*=0.181). However, Sutton et al. [16] presented significantly lower readmission rates of SCT than staples (5.4% vs 7.4%, *P*=0.016). Finally, our meta-analysis demonstrates significantly lower all-cause hospital readmission rates with SCT than staples (*P*=0.03). ② In terms of the wound-related hospital readmission rate, Carli et al. [8] reported that SCT had lower readmission rates than staples (0% vs 1.79%, *P*=0.045). Therefore, we believe that in terms of readmission rates (regardless of all-cause readmission rates or wound-related readmission rates), SCT can reduce the readmission rates of patients after TKA and has better advantages over staples.

Regarding costs and time, ① closure material costs: Takayama et al. [17] reported lower material costs of the SCT than that of the staples ($3 vs $16), but Carli et al. [8] believed the difference oppositely ($63 vs $45). Based on our practical experiences, the different results of two studies are resulted from the different material prices in different countries (Table 1), but we also believe that this difference is not clinically important. ② Skin closure time: there is no article found to compare skin closure time between SCT and staples. But according to our surgical experience, SCT has advantages of simple operation and easy application by just putting the paste with plaster on the wound, which can reduce skin closure time comparing to staples, thus reducing the costs of operating room time and total costs (the average operating expenses of 100 hospitals in the United States is about $62 per minute [20], but it is worth noting that in China the cost of operating room time is not charged). Furthermore, we should also consider to shorten the operation time in order to decrease patients' exposure to narcotic drugs for not only reducing the cost of narcotic drugs but also, more importantly, being safer for the patients. At the same time, though operating room care costs and other costs are also important considerations, all reports have neglected them. ③ Time to postoperative removal: Han Ko et al. [9] reported that the time to removal was shorter in the SCT compared to that of the staples (13.89 ± 0.70 day vs 14.00 ± 0.88 day), which is though not statistically significant (*P*=0.512). It is worth noting that patients feel pain, tension, and fear after the removal of staples [21], as well as suffer from bleeding, infections, and scar formation [22, 23]. Worse still, some patients require local anesthesia before removal [24]. On the contrary, the SCT is easy to be removed and patients feel less fear and pain compared to staples [9]. Therefore, we believe that SCT has the following advantages over staples: ① easy to apply, ② saves time and reduce costs, ③ comfortable for the patients, and ④ easy to remove with no need for others to help.

In terms of VSS and VAS, ① VSS: staples can result in cosmetic problems to patients, and patients will suffer from scar formation lasting about eight weeks on piercing sites after the staples are removed [25]. Han Ko et al. [9] conducted a study to assess VSS that consisted of four parameters: pliability, height, pigmentation, and vascularity, and reported that the VSS on postoperative 90 days was significantly better in the SCT compared to that of the staples. Furthermore, an open, prospective, and controlled randomized clinical study conducted by Parvizi et al. [26] found that the SCT was associated with favorable cosmetic outcomes as compared with other skin closure methods. ② VAS: Han Ko et al. [9] reported that the VAS score on postoperative 1, 3, and 90 days was significantly better in the SCT compared to that of the staples (*P* < 0.05), but there was no significant difference on postoperative 42 and 90 days. Based on our experience, staples closure may increase patients' pain sensations, which could be resulted from several reasons. First, when patients move the knee joint after surgery, relative activities between staples and the skin will be caused, which will increase the pain of patients. Second, then, when the knee joint is moved, patients will feel more pain due to the greater friction force between the dressing and staples. Finally, as staples can be more difficult to remove than SCT, it will increase pain and sometimes require local anesthesia to remove. Therefore, we believe that the use of SCT can reduce patients' early postoperative pain and have a better cosmetic effect.

Thinking about other respects, Takayama et al. [17] showed that the SCT can allow patients to take a shower earlier after surgery than staples (4.4 day vs 4.9 day, *P*=0.0496). Kawakami et al. [27] reported that the satisfaction of patients with SCT exceeded 90%. Sutton et al. [16] reported that SCT could reduce the length of hospital stay for patients (2.8 day vs 3.2 day, *P*=0.002).

In addition, Han Ko et al. [9] reported 3 cases of local skin allergic reaction after the use of SCT, and the local skin allergic reaction was relieved after the removal of SCT, so we believed that SCT might not be applicable to patients with allergic constitution. Even though Carli et al. [8] reported the local blisters after the use of SCT, we still assumed that this phenomenon had no clinical significance.

Our research incorporates all the studies on SCT and staples comparisons throughout all dates and demonstrates the value of the application of SCT in primary TKA. But we acknowledge that this study still has some limitations which need to be further explored: ① this study only includes English literatures, thus bearing a risk of missing some useful research studies; ② the studies included all focus on performance in a short-term follow-up time after TKA, which can impossibly provide long-term efficacy comparisons; and ③ as a newly emerged method, no one has done a relevant statistical analysis. So far, the 4 studies we can collect are a small number of articles presenting an insufficient sample size, making it difficult for us to carry out statistical analysis on other important indexes such as knee range of motion (ROM) and knee society scores (KSS) after operation. Therefore, we need high-quality and large-sample studies to further demonstrate the safety and effectiveness of barbed sutures in the initial TKA application.

## 5. Conclusion

In the primary TKA, compared with the staples, the application of SCT does not increase the incidence of wound complications, but can shorten the closure time, reduce readmission rates, and have a good cosmetic effect. However, the cost and pain need to be further confirmed because of the small sample size included in this study.

## Figures and Tables

**Table 1:** Study characteristics.

First author	Publication	Country	Design	Average age (years)		Group size (knees)		Gender ratio (male/female)		BMI (kg/m2)		Follow-up (months)	Knees
				SCT	Staple	SCT	Staple	SCT	Staple	SCT	Staple		
Sutton et al.	2018	US	CCS	65.1 ± 10	65.5 ± 10.2	971	971	350/621	359/612	NR	NR	3	1942
Takayama et al.	2017	Japan	CS	73.9 ± 6.3	73.8 ± 7.3	38	38	5/33	7/31	25.9 ± 3.2	26.5 ± 4.0	3	76
Carli et al.	2017	USA	CS	60.8 ± 7.38	65.3 ± 9.72	221	1001	100/121	368/629	31.4 ± 6.96	29.6 ± 6.14	3	1222
Han Ko et al.	2017	Korea	CS	68.8 ± 12.3	70.38 ± 10.83	45	45	7/38	11/34	24.90 ± 4.42	24.42 ± 4.28	3	90

NR: not reported; SCT: skin closure tape; BMI: body mass index; CCS: case-control study; CS: cohort study.

## References

[1] Kurtz S., Ong K., Lau E., Mowat F., Halpern M. (2007). Projections of primary and revision hip and knee arthroplasty in the United States from 2005 to 2030. *The Journal of Bone & Joint Surgery*.

[2] Oleske D. M., Bonafede M. M., Jick S., Ji M., Hall J. A. (2014). Electronic health databases for epidemiological research on joint replacements: considerations when making cross-national comparisons. *Annals of Epidemiology*.

[3] Kurtz S. M., Lau E., Ong K. (2009). Future young patient demand for primary and revision joint replacement: national projection from 2010–2030. *Clinical Orthopaedics and Related Research*.

[4] Patel R. M., Cayo M., Patel A., Albarillo M., Puri L. (2012). Wound complications in joint arthroplasty: comparing traditional and modern methods of skin closure. *Orthopedics*.

[5] Zhang W., Xue D. T., Yin H. F. (2016). Barbed versus traditional sutures for wound closure in knee arthroplasty: a systematic review and meta-analysis. *Scientific Reports*.

[6] Kim K. Y., Anoushiravani A. A., Long W. J., Vigdorchik J. M., Fernandez-Madrid I., Schwarzkopf R. (2017). A meta-analysis and systematic review evaluating skin closure after total knee arthroplasty-what is the best method?. *The Journal of Arthroplasty*.

[7] Krishnan R., Macneil S. D., Malvankar-Mehta M. S. (2016). Comparing sutures versus staples for skin closure after orthopardic surgery: systematic review and meta-analysis. *BMJ Open*.

[8] Carli A. V., Spiro S., Barlow B. T., Haas S. B. (2017). Using a non-invasive secure skin closure following total knee arthroplasty leads to fewer wound complications and no patient home care visits compared to surgical staples. *The Knee*.

[9] Han Ko J., Yang L. H., Ko M. S., Kamolhuja E., Park K. K. (2017). Do zip-type skin-closing devices show better wound status compared to conventional staple devices in total knee arthroplasty?. *International Wound Journal*.

[10] Ge L., Tian J.-H., Li Y.-N. (2018). Association between prospective registration and overall reporting and methodological quality of systematic reviews: a meta-epidemiological study. *Journal of Clinical Epidemiology*.

[11] Tian J., Zhang J., Ge L., Yang K., Song F. (2017). The methodological and reporting quality of systematic reviews from China and the USA are similar. *Journal of Clinical Epidemiology*.

[12] Yao L., Sun R., Chen Y.-L. (2016). The quality of evidence in Chinese meta-analyses needs to be improved. *Journal of Clinical Epidemiology*.

[13] Wang X., Chen Y., Yao L. (2018). Reporting of declarations and conflicts of interest in WHO guidelines can be further improved. *Journal of Clinical Epidemiology*.

[14] Yan P., Yao L., Li H. (2019). The methodological quality of robotic surgical meta-analyses needed to be improved: a cross-sectional study. *Journal of Clinical Epidemiology*.

[15] Pieper D., Buechter R. B., Li L., Prediger B., Eikermann M. (2015). Systematic review found AMSTAR, but not R(evised)-AMSTAR, to have good measurement properties. *Journal of Clinical Epidemiology*.

[16] Sutten N., Schmitz N.-D., Stephen S. (2018). Economic and clinical comparison of 2-octyl cyanoacrylate/polymer mesh tape with skin staples in total knee replacement. *Journal of Wound Care*.

[17] Takayama S., Yamamoto T., Tsuchiya C., Noguchi H., Sato J., Ishii Y. (2017). Comparing Steri-Strip and surgical staple wound closures after primary total knee arthroplasties. *European Journal of Orthopaedic Surgery & Traumatology*.

[18] Gaine W. J., Ramamohan N. A., Hussein N. A., Hullin M. G., McCreath S. W. (2000). Wound infection in hip and knee arthroplasty. *The Journal of Bone and Joint Surgery*.

[19] Meena S., Gangary S., Sharma P., Chowdhury B. (2015). Barbed versus standard sutures in total knee arthroplasty: a meta-analysis. *European Journal of Orthopaedic Surgery & Traumatology*.

[20] Macario A. (2010). What does one minute of operating room time cost?. *Journal of Clinical Anesthesia*.

[21] Hlubek R., Walde P., Kana J. (2014). Metal staples versus conventional suture for wound closure in total knee arthroplasty. *Acta Chir Orthop Tranmatol Cech*.

[22] Singh B., Mowbray M. A. S., Nunn G., Mearns S. (2006). Closure of hip wound, clips or subcuticular sutures: does it make a difference?. *European Journal of Orthopaedic Surgery & Traumatology*.

[23] Khan R. J., Fick D., Yao F. (2006). A comparison of three methods of wound closure following arthroplasty: a prospective, randomized, controlled trial. *The Journal of Bone and Joint Surgery*.

[24] Tseng T.-H., Jiang C.-C., Fu S.-H., Lee T.-L., Chuang Y.-H., Chiang H. (2017). Topical anesthesia for staple removal from surgical wounds on the knee: a prospective, double-blind, randomized trial. *Journal of Surgical Research*.

[25] Shetty A., Kumar V., Morgan-Hough C., Georgeu G., James K., Nicholl J. (2004). Comparing wound complication rates following closure of hip wounds with metallic skin staples or subcuticular vicryl suture: a prospective randomised trial. *Journal of Orthopaedic Surgery*.

[26] Parvizi D., Friedl H., Schintler M. V. (2013). Use of 2-octyl cyanoacrylate together with a self-adhering mesh (Dermabond™ Prineo™) for skin closure following abdominoplasty: an open, prospective, controlled, randomized, clinical study. *Aesthetic Plastic Surgery*.

[27] Kawakami H., Setoyama T., Uchiyamada S., Tominaga H., Tsuneyoshi Y., Komiya S. (2012). Usefulness of steri-strips&reg; and Dermabond® in orthopedic surgery. *Orthopedics & Traumatology*.

